# IMPROVED FUNCTIONAL ORAL INTAKE AND EXERCISE TRAINING ATTENUATE DECLINE IN AEROBIC CAPACITY FOLLOWING CHEMORADIOTHERAPY IN PATIENTS WITH OESOPHAGEAL CANCER

**DOI:** 10.2340/jrm.v56.25906

**Published:** 2024-10-18

**Authors:** Shu-Chun HUANG, Lan-Yan YANG, Yin-Kai CHAO, Wei-Yang CHANG, Ya-Tzu TSAO, Chuan-Yi CHOU, Ching-Chung HSIAO, Chien-Hung CHIU

**Affiliations:** 1 Department of Physical Medicine and Rehabilitation, New Taipei Municipal Tucheng Hospital, Chang Gung Memorial Hospital, New Taipei City, Taiwan; 2 Department of Physical Medicine & Rehabilitation, Chang Gung Memorial Hospital, Linkou, Taoyuan, Taiwan; 3 College of Medicine, Chang Gung University, Kwei-Shan, Tao-Yuan County, Taiwan; 4 Clinical Trial Center, Chang Gung Memorial Hospital, Taoyuan, Taiwan; 5 Division of Clinical Trial, Taichung Veterans General Hospital, Taichung, Taiwan; 6 Division of Thoracic Surgery, Chang Gung Memorial Hospital, Linkou, Taoyuan, Taiwan; 7 Department of Medical Nutrition Therapy, Chang Gung Memorial Hospital, Linkou, Taoyuan, Taiwan; 8 Department of Nephrology, New Taipei Municipal Tucheng Hospital, Chang Gung Memorial Hospital, New Taipei City, Taiwan

**Keywords:** chemoradiotherapy, fitness, prehabilitation, swallowing, nutrition

## Abstract

**Purpose:**

To investigate the impact of chemoradiotherapy on the physical fitness of patients with oesophageal cancer, and the clinical factors influencing it.

**Method:**

A total of 67 participants successfully completed the study, with 18 of them engaging in supervised, in-hospital aerobic training at moderate intensity for a minimum of 20 sessions. Cardiopulmonary exercise testing, hand grip strength, body composition assessed via bioelectrical impedance analysis, patient-generated subjective global assessment, albumin, and the Functional Oral Intake Scale (FOIS) were evaluated before chemoradiotherapy and 6–8 weeks after its completion.

**Result:**

Among the participants, cardiopulmonary fitness, hand grip strength, and phase angle of BC-BIA declined during chemoradiotherapy. Before and after chemoradiotherapy, V̇O_2peak_ was 19.6 ± 4.4 and 17.4 ± 3.9 mL/min/kg respectively. The improvement in FOIS during chemoradiotherapy showed a positive correlation with changes in aerobic capacity. Additionally, exercise training was associated with attenuating the decline in aerobic capacity.

**Conclusion:**

Physical fitness deteriorated in patients with oesophageal cancer following chemoradiotherapy. Improvement in dysphagia helps maintain aerobic capacity. Additionally, exercise training has the potential to mitigate the decline. This discovery can serve as a reference for enhancing holistic care for patients with oesophageal cancer.

## LAY ABSTRACT

After treatment with chemoradiotherapy, patients with oesophageal cancer often find that their aerobic fitness, hand grip strength, and certain body composition measurements like phase angle decrease. These effects can last for at least 6–8 weeks after treatment. Interestingly, the present study suggests that as swallowing function gets better, so does aerobic capacity, which is a new finding. Doing structured exercise during chemoradiotherapy, such as 20 sessions of moderate-intensity workouts lasting 30 min each, might also stop aerobic capacity from dropping too much.

Oesophageal cancer constitutes a highly lethal ailment with a poor long-term survival rate. However, since the implementation of multimodality therapy, the prognosis for oesophageal cancer patients has shown significant improvement (1, 2). The integration of various therapeutic approaches, including chemoradiotherapy (CRT), has played a pivotal role in enhancing the outcomes of advanced oesophageal cancer. In the case of locally advanced oesophageal cancer, the standard therapeutic approach involves neoadjuvant CRT (nCRT) followed by oesophagectomy, with curative intent for patients deemed suitable for surgery (3, 4). On the other hand, definitive CRT (dCRT) stands as an alternative curative treatment strategy for patients diagnosed with cervical oesophageal cancer or those who are not suitable candidates for surgical interventions (3, 5).

Nonetheless, CRT could have an unfavourable effect on physical fitness, encompassing cardiopulmonary fitness, muscle strength, and body composition (6–9). Literature regarding the impact of nCRT on physical fitness is inconsistent. Most studies showed that exercise capacity declined during nCRT (8–13). The regression persists even immediately before oesophagectomy, which is about 4–6 weeks after completion of nCRT (8, 11, 12). On the other hand, 2 studies reported that exercise capacity was not changed after nCRT (14, 15). The discrepant result may result from relatively small sample size, which ranged from 21 to 56 participants in the aforementioned 8 studies. Among these, 2 studies further showed that prehabilitation attenuated the declining trend of exercise capacity during nCRT (8, 9). However, only 6-min-walk distance (6MWD), a submaximal testing, was used as the measure of cardiopulmonary fitness. Moreover, factors that influence the change in physical fitness during CRT have not been investigated.

Accordingly, in the present study, to address the contradictory findings, a larger sample size was implemented to investigate whether CRT has a negative impact on physical fitness. Cardiopulmonary exercise testing by gas analysis (CPET), the gold-standard measure of cardiopulmonary fitness, was used to determine the magnitude of the impact of CRT on aerobic capacity. In-hospital supervised exercise training was facilitated. Hand grip strength (HGS), body composition by bioelectrical impedance analysis (BC-BIA), functional oral intake scale (FOIS), and nutritional status were also evaluated. We hypothesized that physical fitness would deteriorate during CRT, and that improvements in nutritional status, swallowing function, and exercise training could attenuate this decline.

## METHODOLOGY

### Participants and design

The patients were evaluated for eligibility based on the following criteria: (*i*) age 20 years or older, (*ii*) a diagnosis of oesophageal cancer, and (*iii*) having a planned course of concurrent chemoradiotherapy (CRT) with curative intent, including oesophagectomy after neoadjuvant chemoradiotherapy (nCRT) and definitive chemoradiotherapy (dCRT). Exclusion criteria included comorbid medical, physical, and mental conditions that contraindicate exercise, acute or unstable cardiac conditions (such as unstable angina or symptomatic severe aortic stenosis), disabling orthopaedic or neuromuscular diseases, and dementia (16).

A total of 138 patients diagnosed with oesophageal cancer who underwent oesophagectomy after receiving neoadjuvant chemoradiotherapy (nCRT) or definitive chemoradiotherapy (dCRT) at Chang Gung Memorial Hospital in Linkou, Taiwan, between February 2020 and February 2023, were screened. After excluding individuals who had contraindications for exercise testing or those who could not ride on the stationary cycle ergometer (*n* = 19), and those who declined to participate in the study (*n* = 47), a total of 72 patients were eligible for inclusion in our analysis.

The assessment of physical fitness and nutrition occurred at 2 specific time points: initially at baseline and 6–8 weeks after the completion of chemoradiotherapy (CRT). Every patient received comprehensive explanations regarding the exercise training programme as an integral part of the protocol. The participants decided whether to join the exercise training programme based on their own will. The study protocol was approved by the Institutional Review Board of Chang Gung Memorial Hospital.

### Exercise training programme

The exercise protocol was a hospital-based supervised programme comprising aerobic training by cycle ergometer. The participants attended the rehabilitation centre 5 times a week to undergo exercise therapy, typically scheduled on the day before or after their radiotherapy sessions. Exercise sessions might be temporarily halted on the day or for several days following chemotherapy if patients were unable to tolerate training. The intensity was set initially at ventilatory anaerobic threshold and gradually titrated up to respiratory compensatory point based on the breath-by-breath cardiopulmonary exercise testing. The duration was 30 min per session plus 5-min warm-up and 5-min cool-down. Those who decided to participate in the exercise training programme were required to complete a minimum of 20 sessions in total.

### Cardiopulmonary exercise testing

A symptom-limited incremental exercise test was performed in the upright position on a calibrated bicycle ergometer (Ergoselect 150P; ergoline GmbH, Bitz, Germany) to assess aerobic fitness and haemodynamic function. CPET was performed 2–4 h after a light meal. It began with 2 min of rest and 1 min of warm-up at 10 W, followed by a ramp increase of 10 W every min, until exhaustion. Minute ventilation (V̇_E_), oxygen consumption (V̇O_2_), and carbonic dioxide production (V̇CO_2_) were measured breath-by-breath using a computer-based system (MasterScreen CPX, Cardinal Health, Halberstadt, Germany). The data were averaged every 15 s. Four parameters were employed to assess the ventilation efficiency: (*i*) EqCO_2nadir_ refers to the smallest value of ventilatory equivalent for CO_2_ (V̇_E_ /V̇CO_2_) during incremental exercise testing (17); (*ii*) oxygen uptake efficiency slope (OUES) was derived from the slope of V̇O_2_ vs natural logarithm of V̇_E_, OUES is an estimation of the efficiency of ventilation with respect to V̇O_2_, with a greater slope indicating higher oxygen uptake efficiency (18); (*iii*) the V̇_E_ –V̇CO_2_ slope was calculated as Y mX+b, where Y is V̇_E_, X is V̇CO_2_, and m is the slope. V̇_E_ and V̇CO_2_ were acquired from the initiation of exercise to the peak values. A more horizontal slope suggests better ventilation efficiency (19). Heart rate (HR) was determined from the R–R interval on a 12-lead electrocardiogram, arterial pressure was measured using an automatic blood pressure system (Tango, SunTech Medical, Eynsham, UK), and arterial O_2_ saturation was monitored using finger pulse-oximetry (model 9500, Nonin Onyx, Plymouth, MA, USA). The exercise test was terminated using the following criteria: (1) the subject could not keep up with the pedalling frequency to 50 rpm; (2) the participant reached volitional fatigue and was requested to end the test; (3) the participant’s peak V̇O_2_ plateaued or decreased despite the continuation of exercise, or (4) an adverse cardiovascular event was observed. Ventilatory anaerobic threshold (VAT) was determined primarily by the V-slope method and verified based on ventilatory criteria as follows: (*i*) departure from linearity for V̇CO_2_ against V̇O_2_, (*ii*) the V̇_E_-V̇O_2_ ratio increased without a corresponding increase in the V̇_E_–V̇CO_2_ ratio and (*iii*) end-tidal tensions of oxygen increases without a corresponding decrease in end-tidal tensions of carbon dioxide (20). VAT was identified by 2 independent reviewers.

### Body composition

Whole-body composition was determined using InBody s10 (Seoul, Korea) and by measuring the electrical resistance to 4 different frequencies (5, 50, 250, and 500 kHz) (21, 22). Each participant remained seated upright on a non-conductive chair throughout the entire testing duration. The sensors measuring electrical resistance were placed at the level of each body segment following the manufacturer instructions. To undergo this 10-min procedure, participants were instructed to fast for 2 h prior to the test. Appendicular skeletal muscle index (ASMI), body cell mass (BCM), fat free mass (FFM), lean body mass (LBM), phase angle (PA), and body fat percentage (BF) were recorded.

### Nutrition assessment

The nutrition assessments comprised bodyweight (BW), albumin blood levels, and the Patient-Generated Subjective Global Assessment (PGSGA). PGSGA is a nutritional evaluation tool designed for cancer patients, with a higher score indicating a greater risk of malnutrition (23). The PG-SGA assessments were conducted by a specialized dietitian.

### Functional oral intake scale

The Functional Oral Intake Scale (FOIS) is a validated tool used to evaluate the functional oral intake status of individuals with dysphagia (24, 25). FOIS categorizes individuals into 7 levels, reflecting their capacity to safely and effectively consume food and liquids by mouth. 1 = No oral intake; 2 = Tube dependent with minimal/inconsistent oral intake; 3 = Tube supplements with consistent oral intake; 4 = Total oral intake of a single consistency; 5 = Total oral intake of multiple consistencies requiring special preparation; 6 = Total oral intake with no special preparation, but must avoid specific foods, 7 = Total oral intake with no restrictions.

### Statistics

The data were expressed as the mean ± standard deviation and were analysed using IBM SPSS Statistics 22.0 (IBM Corp, Armonk, NY, USA). In the comparison between participants who underwent supervised exercise training with those who did not (received usual care), the χ^2^ test was used to analyse the correlation between categorical variables, while the independent *t*-test was used for continuous variables. A paired t-test was employed to compare the pre- and post-CRT statuses. Additionally, univariate and multivariate forward linear stepwise regression analyses were conducted to identify parameters correlating with the change in V̇O_2peak_ (mL/min) (Δ V̇O_2peak_ = V̇O_2peak_ at post-CRT - V̇O_2peak_ at pre-CRT). Independent variables with *p*-value < 0.1 in the univariate analysis were included in the multivariate linear stepwise regression. “Δ” represents the difference between the post-CRT value and the pre-CRT status value. The criterion for significance was *p*-value < 0.05.

## RESULTS

Five dropped out due to their reluctance to return of their own volition. Ultimately, 67 participants successfully completed the study. Forty-eight patients received nCRT and 19 patients underwent dCRT. Eighteen participated in the supervised exercise training programme (Table I). Among them, no exercise-related adverse events were reported and the adherence rate was 100%. No significant difference was found in the basic data between participants who underwent supervised exercise training and those who did not (received usual care) (Table I).

**Table I:** Basic information

Variables	Overall (*n* = 67)	Training (*n* = 18)	UC (*n* = 49)	*p*-value
Age, year, mean ± SD	57 ± 8	56 ± 8	58 ± 7	0.282
Body height, cm, mean ± SD	168.3 ± 6.5	170.1 ± 7.0	167.6 ± 6.3	0.157
Bodyweight, kg, mean ± SD	65.5 ± 12.6	68.9 ± 14.0	64.2 ± 11.9	0.175
BMI, kg/m^2^, mean ± SD	32.0 ± 3.7	23.7 ± 3.8	22.8 ± 3.6	0.384
Gender, *n* (%)				0.384
Male	65 (97)	18 (100)	47 (96)	
Female	2 (3)	0 (0)	2 (0)	
Clinical staging, *n* (%)				0.807
II	1 (1)	0 (0)	1 (2)	
III	50 (75)	14 (78)	36 (74)	
IV	16 (24)	4 (22)	12 (24)	
Pathology, *n* (%)				0.931
SCC	63 (94)	17 (94)	46 (94)	
Adenocarcinoma	4 (6)	1 (6)	3 (6)	
RT dose, *n* (%)				0.321
< 4500 cGY	17 (25)	3 (17)	14 (29)	
≤4500 cGY	50 (75)	15 (83)	35 (71)	
CT regimen, *n* (%)				0.153
Cisplatin+5-fluorouracil	20 (30)	3 (17)	17 (35)	
Carboplatin+paclitaxel	47 (70)	15 (83)	32 (65)	
CRT goal, *n* (%)				0.603
Neoadjuvant	49 (73)	14 (78)	35 (71)	
Definitive	18 (27)	4 (22)	14 (29)	
CRT response, *n* (%)				0.719
Yes	62 (93)	17 (94)	45 (92)	
No	5 (7)	1 (6)	4 (8)	
Subsequent oesophagectomy, *n* (%)				0.872
Yes	42 (62)	11 (61)	31 (63)	
No	25 (39)	7 (39)	18 (37)	

SD: standard deviation; BMI: body mass index; UC: usual care; CT: chemotherapy; CRT: chemoradiotherapy; RT: radiotherapy.

All the cardiopulmonary fitness-related parameters, whether derived from peak or submaximal status, showed a decline in the post-CRT condition compared with the pre-CRT, persisting even 6–8 weeks after the completion of CRT (Table II). Specifically, VO_2peak_ decreased from 19.6 ± 4.4 to 17.4 ± 3.9 mL/min/kg and HGS also declined at 41.7 ± 7.9 vs 39.6 ± 8.6 kg. Regarding BC-BIA, the PA exhibited deterioration in all 4 limbs and the trunk following CRT. However, ASMI, BCM, FFM, LBM, and BF remain unchanged. In terms of nutritional status, PGSGA showed improvement. Conversely, albumin levels exhibited a declining trend with marginal significance (Table II).

**Table II:** Physical fitness, body composition, and nutrition status before and after chemoradiotherapy (CRT)

Factor	Unit	pre-CCRT	post-CCRT	*p*-value
VO_2peak_	mL/min	1,259.1 ± 297.9	1,112.3 ± 268.1	< 0.001
VO_2peak_	mL/min/kg	19.6 ± 4.4	17.4 ± 3.9	< 0.001
Predicted VO_2peak_	%	73 ± 15	66.3 ± 12.0	< 0.001
WR_peak_	watt	100 ± 25	92.9 ± 23.3	< 0.001
AT	mL/min	705.6 ± 175.7	643.6 ± 131.2	0.001
AT	mL/min/kg	11.00 ± 2.81	10.17 ± 2.19	0.004
WR_VAT_	watt	43 ± 15	37 ± 13	0.002
EqO_2_@AT		29.0 ± 5.0	31.2 ± 5.4	< 0.001
EqCO_2nadir_		30.2 ± 4.6	32.5 ± 5.4	< 0.001
OUES		629 ± 139	571 ± 125	< 0.001
V_E_–VCO_2_ slope		30.4 ± 5.2	33.0 ± 6.1	< 0.001
HGS	kg	41.7 ± 7.9	39.6 ± 8.6	0.002
ASMI	kg/m^2^	7.63 ± 0.97	7.64 ± 1.04	0.796
BCM	kg	32.7 ± 5.5	32.6 ± 5.7	0.639
FFM	kg	49.9 ± 8.6	50.4 ± 8.6	0.260
LBM	kg	46.9 ± 8.8	47.8 ± 8.2	0.121
PARA		6.0 ± 0.8	5.5 ± 0.8	< 0.001
PALA		5.9 ± 0.8	5.5 ± 0.9	< 0.001
PATR		8.6 ± 1.7	8.0 ± 1.5	0.014
PARL		6.2 ± 0.9	5.6 ± 1.0	< 0.001
PALL		5.9 ± 0.9	5.4 ± 1.0	< 0.001
Body fat	%	23.0 ± 6.7	22.3 ± 6.4	0.191
BW	kg	65.5 ± 12.6	65.4 ± 11.9	0.862
PGSGA		6.2 ± 3.3	3.6 ± 3.3	< 0.001
FOIS		5.5 ± 1.7	5.7 ± 1.6	0.152
Albumin	g/dL	4.25 ± 0.36	4.07 ± 0.44	0.054

ASMI: appendicular skeletal muscle index; AT: ventilatory anaerobic threshold; BCM: body cell mass; BW: bodyweight; EqO_2_: ventilatory equivalent for O_2_; EqCO_2_: ventilatory equivalent for CO_2_, FFM: fat free mass; FOIS: functional oral intake scale; HGS: hand grip strength, LA: left arm; LBM: lean body mass; LL: left leg; OUES: oxygen uptake efficiency slope; PA: phase angle; PGSGA: patient-generated-subjective global assessment; RA: right arm; RER: respiratory exchange ratio; RL: right leg; TR: trunk; V̇O_2_: oxygen consumption; V̇CO_2_: CO_2_ production; V̇_E_: ventilation; WR: work rate. *p < 0.05, paired t- test, two-tailed.

To identify the parameters correlated with Δ V̇O_2peak_ (mL/min), those with a *p*-value less than 0.1 in the univariate linear analysis were included in the multivariate linear stepwise regression. These parameters encompass exercise training, preV̇O_2peak_ (mL/min), preVO_2peak_ (mL/min/kg), preAT (mL/min), preEqO_2_@AT, preOUES, Δ HGS (kg), Δ FOIS, and Δ ASMI (kg/m^2^). When they were entered into the multivariate forward linear stepwise regression model, 3 of them exhibited a significant regression coefficient: preV̇O_2peak_ (mL/min), exercise training, and Δ FOIS (Table III).

**Table III:** Univariate and multivariate forward linear stepwise regression on Δ V̇O_2peak_ (mL/min)

Parameter	Univariate model (*n* = 67)		Multivariate model (*n* = 67)	
	Model coefficient B (95% CI)	*p*-value	Model coefficient (95% CI)	*p*-value
Sex	–151.557 (–460.863, 157.749)	0.331		
Age (year)	–2.315 (–9.344, 5.073)	0.556		
PreBMI (kg/m^2^)	–11.281 (–25.579, 3.017)	0.120		
PreBW (kg)	–1.85 (–6.073, 2.374)	0.385		
RTO dose	15.019 (–106.774, 136.813)	0.806		
CT regimen	–15.870 (–133.440, 101.700)	0.788		
CRT goal	–1.474 (–121.093, 118.144)	0.980		
CRT response	–31.01 (–226.573, 164.553)	0.752		
Subsequent oesophagectomy	–45.181 (–153.411, 63.049)	0.407		
Exercise training	–117.059 (–233.111, 1.008)	0.048	–148.552 (–244.736, –52.368)	0.003
PreV̇O_2peak_ (mL/min)	–3.22 (–4.61, –0.183)	< 0.001	–0.339 (–0.467, –0.211)	< 0.001
PreVO_2peak_ (mL/min/kg)	–18.885 (–28.626, –9.144)	< 0.001		
PreAT (mL/min)	–3.90 (–0.678, –0.102)	0.009		
PreAT (mL/min/kg)	–14.715 (–33.368, 3.938)	0.120		
PreEqO_2_@AT	10.389 (–0.952, 21.731)	0.072		
PreEqCO_2nadir_	7.998 (–2.557, 18.553)	0.135		
PreOUES	–0.623 (–0.974, –0.271)	0.001		
PreV’_E_-V’CO_2_ slope	4.399 (–5.869, 14.667)	0.395		
PreHGS (kg)	–2.299 (–9.033, 4.434)	0.498		
Δ HGS (kg)	10.685 (0.018, 21.352)	0.050		
PreBW (kg)	–1.85 (–6.073, 2.374)	0.385		
PrePGSGA	–1.047 (–17.163, 15.069)	0.897		
Δ PGSGA	–8.347 (–19.231, 2.538)	0.130		
PreFOIS	–7.654 (–25.573, 10.264)	0.397		
Δ FOIS	63.997 (27.720, 100.274)	0.001	41.060 (9.596, 72.524)	0.011
PreAlbumin (g/dL)	–15.347 (–71.281, 40.587)	0.586		
Δ Albumin (g/dL)	36.568 (–86.511, 159.648)	0.555		
PreASMI (kg/m^2^)	–19.248 (–51.839, 13.344)	0.243		
Δ ASMI (kg/m^2^)	114.412 (–1.956, 230.781)	0.054		
PreBCM (kg)	–3.270 (–10.050, 3.509)	0.339		
Δ BCM (kg)	–9.522 (–23.688, 4.644)	0.184		
PreBCM/BW	–57.814 (–590.022, 474.393)	0.829		
Δ BCM/BW	–674.841 (–1707.248, 516.943)	0.196		
PrePARA	–20.858 (–61.995, 20.279)	0.315		
Δ PARA	–2.291 (–11.003, 6.420)	0.601		
PrePALA	–26.249 (–68.351, 15.853)	0.218		
Δ PALA	49.789 (–41.816, 141.394)	0.281		
PrePATR	–3.283 (–27.251, 20.686)	0.785		
Δ PATR	6.774 (–30.227, 43.775)	0.716		
PrePARL	–20.858 (–61.995, 20.279)	0.315		
Δ PARL	25.891 (–52.187, 003.969)	0.510		
PrePALL	–26.249 (–68.351, 15.853)	0.218		
Δ PALL	38.432 (–38.422, 115.286)	0.321		
Prebody fat (%)	–4.007 (–10.871, 2.857)	0.248		
Δ body fat (%)	1.374 (–12.010, 14.757)	0.838		

Dependent variable = Δ V̇O_2peak_ = VO_2peak_ at post-CRT –V̇O_2peak_ at pre-CRT (mL/min).

“Pre” indicates before CRT; “Δ” represents the difference between the post-CRT value and the pre-CRT status value. Other abbreviations are as described in Tables I and II.

**Fig. 1:**
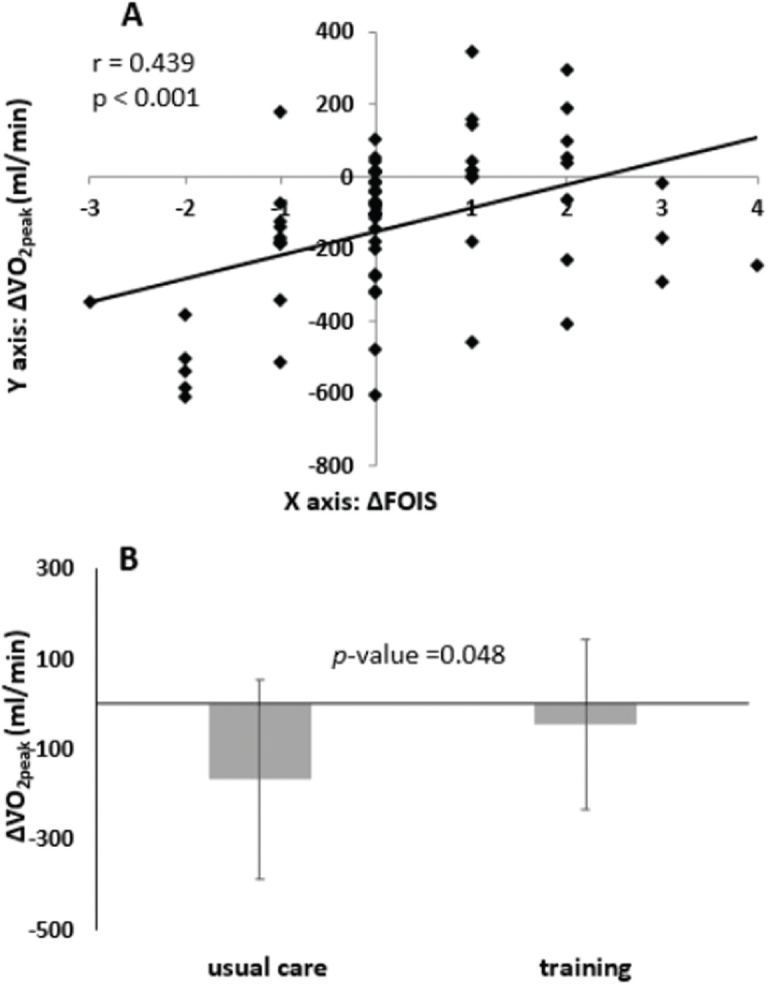
(A) An improvement in Δ FOIS is associated with a positive change in ΔV̇O_2peak_. (B) Exercise training correlates with a decline in Δ V̇O_2peak_ during CRT. Usual care vs training V̇O_2peak_ = –168 ± 222 vs –47 ± 189 (mL/min); –2.4 ± 3.4 vs –1.0 ± 2.4 (mL/min/kg).

Fig. 1A illustrates that an improvement in Δ FOIS correlates with a positive change in Δ V̇O_2peak_. After CRT, among the minority (19/67, 28%) who experienced an increase in V̇O_2peak_, nearly all demonstrated improvement in Δ FOIS (18/19, 95%, first quadrant), with only 1 exception (1/19, second quadrant). Fig. 1B demonstrates that exercise training is associated with a reduction in the declining trend following CRT. Additionally, a higher preV̇O_2peak_ is correlated with a larger negative change in Δ is _2peak_.

It is worth mentioning that, in this cohort, the following factors do not show an association with Δ V̇O_2peak_ (mL/min): radiotherapy dose (> 4500 vs ≤ 4500 cGy), CT regimen (cisplatin+5-fluorouracil vs carboplatin+paclitaxel), CRT goal (neoadjuvant vs definitive), CRT response and whether subsequent oesophagectomy was performed.

## DISCUSSION

The primary experimental findings can be summarized as follows. In patients with oesophageal cancer, cardiopulmonary fitness significantly declined following CRT, persisting for at least 6–8 weeks after the completion of CRT. The magnitude of VO_2peak_ decrease (19.6 ± 4.4 vs 17.4 ± 3.9 mL/min/kg) in the present cohort is clinically significant. Interestingly, the improvement in FOIS during CRT is positively correlated with changes in cardiopulmonary fitness, a novel finding proposed by the present study. Following CRT, among the minority (28%) who experienced an increase in V̇O_2peak_, nearly all demonstrated improvement in Δ FOIS (95%). Meanwhile, the decline in aerobic capacity could potentially be attenuated by structured exercise training during CRT, which was well received by the participants. Additionally, CRT has an adverse impact on patients’ strength, as evidenced by HGS. BC-BIA indicated no significant changes in ASMI, BCM, FFM, or LBM after CRT. However, the phase angle for both the trunk and all 4 extremities showed an average decrease ranging from 0.4 to 0.6 degrees. Thus, PA is the most sensitive marker among the BC-BIA measurement to monitor the patient’s overall health and nutritional status in the oesophageal patients receiving CRT.

### Influence of CRT

The literature on how cardiopulmonary fitness changes after CRT displays inconsistencies. While most studies suggested a decrease (8–13), a minority reported no significant change (14, 15). The current experimental findings align with the majority of previous research results. Furthermore, the current study demonstrates that recovery of aerobic capacity does not occur within 6–8 weeks after completing CRT. Consistent with these findings, prior studies also indicated that the observed decrease in AT, V̇O_2peak_ or 6MWD following nCRT does not spontaneously improve after 4 weeks or 4–6 weeks following the completion of nCRT (8, 11, 12).

Elements from 3 domains (chemotherapy, radiotherapy, and cancer-related factors) can have a negative impact on physical fitness. In the present investigation, 2 combinations of chemotherapeutic agents were used: cisplatin+5-fluorouracil and carboplatin+paclitaxel. These can result in diminished cardiorespiratory fitness, primarily attributed to their impact on the cardiovascular and respiratory systems (10). Fatigue and anaemia are also common side effects in these 4 chemotherapy medications in the present study, ranging from 11~90% based on Micromedex®. Fluorouracil also has common side effect of anorexia. These treatments also influence cellular and mitochondrial metabolism. Cisplatin and paclitaxel were reported to alter the function of mitochondria, followed by disruption of respiratory chain function and increased production of reactive oxygen species (26). In addition, radiotherapy can result in radiation pneumonitis (27). Other than chemoradiotherapy effects, cancer-related factors such as poor nutrition and cancer cachexia may also contribute to deterioration of physical fitness.

Phase angle has been used as a marker of nutritional status, muscle mass and function, and cell integrity (28). Previous studies have shown a decrease in PA following chemotherapy in non-small-cell lung cancer (29) and breast cancer (30, 31). To the best of our knowledge, this study is the first to demonstrate that PA decreases following CRT in patients with oesophageal cancer, both in the trunk and in all 4 extremities. The average reduction magnitude ranges from 0.4 to 0.6 degrees. Conversely, no changes were noted in ASMI, BCM, FFM, or LBM, indicating that PA may serve as a more sensitive marker.

### Functional Oral Intake Scale

The present finding indicates a significant association between Δ FOIS and Δ V̇O_2peak_. Improvement in swallowing function benefits physical fitness. It is important to note that other related indicators, such as CRT response, PGSGA, albumin, and BW, do not exhibit a similar correlation. Hence, FOIS is a convenient and valuable scale for evaluating swallowing function in oesophageal cancer patients. In a cross-sectional study by Matsuda et al., self-efficacy tended to increase as the FOIS improved in cancer patients (32). To the best of our knowledge from the literature search, this study is the first to utilize FOIS for quantifying clinical outcomes of dysphagia and demonstrating its value in oesophageal cancer patients (33–35).

### Exercise training

Two published randomized clinical trials (RCT) have investigated exercise training during nCRT in patients with oesophageal cancer. In the first study (8), when compared with the control group (*n* = 25), prehabilitation (*n* = 26) effectively prevented a decline in the 6MWD. Prehabilitation encompassed aerobic moderate continuous training 3 times weekly, resistance training, and nutrition optimization. The median length of prehabilitation was 36 days (IQR 17–73 days). In the second RCT (9), a walk-and-eat intervention was employed, where participants in the intervention group completed an average of 8.4 ± 3.6 sessions of moderate-intensity walking before and after chemoradiation therapy over a span of 4–5 weeks of nCRT. Likewise, this intervention mitigated the decline in 6MWD and HGS when compared with the control group. In the 2 aforementioned studies, 6MWD, a submaximal test, was utilized to assess aerobic capacity. The result of the current study is similar to the 2 previous studies, but instead employed CPET, the gold standard for measuring cardiopulmonary fitness. BC-BIA and HGS were also incorporated to evaluate physical fitness comprehensively. Moreover, exercise training might also benefit postoperative morbidity (36). Future studies on multimodal prehabilitation are necessary to refine optimal programmes for patients with oesophagogastric cancer.

### Nutrition

Malnutrition is common for oesophageal cancer patients due to a combination of mechanical obstruction and cancer cachexia. In the current cohort, PGSGA showed significant improvement following CRT, whereas there seemed to be a borderline decrease in albumin levels. This phenomenon is likely attributable to the nature of PGSGA, a patient-generated subjective score; a reduction in the score indicates a reduction in the risk of malnutrition. On the other hand, a decrease in albumin level does not necessarily indicate malnutrition. It has been reported that serum albumin concentration is not a useful marker for malnutrition (37). In our case, the oxidative and inflammatory stress generated by CRT can deplete serum albumin, leading to a decrease in albumin level (38). Therefore, PGSGA may improve more rapidly during CRT. In contrast, given albumin’s 22-day half-life and the impact of CRT, its blood levels may take some time to recover.

### Limitation

The study has a limitation as it is a prospective observational study; the patients opted to participate in the exercise training programme, introducing potential selection bias when evaluating the impact of exercise training. However, the primary objective of the current study was to investigate diverse clinical factors associated with changes in aerobic capacity following CRT, going beyond the specific focus on exercise training alone.

### Conclusion

Physical fitness of patients with oesophageal cancer, including cardiopulmonary fitness, HGS, and phase angle of body composition by bioelectrical impedance analysis, declined following CRT. The improvement in dysphagia during CRT is significantly positively correlated with changes in aerobic capacity. Additionally, the decline in aerobic capacity could potentially be attenuated by structured exercise training. This discovery can serve as a reference for enhancing holistic care for patients with oesophageal cancer focused on improving healthcare quality.
